# Effective oxygen metabolism-based prognostic signature for colorectal cancer

**DOI:** 10.3389/fonc.2023.1072941

**Published:** 2023-02-09

**Authors:** Yonghui Yuan, Zhong-guo Zhang, Bin Ma, Pengfei Ji, Shiyang Ma, Xun Qi

**Affiliations:** ^1^ Liaoning Cancer Hospital & Institute, Clinical Research Center for Malignant Tumor of Liaoning Province, Cancer Hospital of China Medical University, Shenyang, Liaoning, China; ^2^ Large-Scale Data Analysis Center of Cancer Precision Medicine, Cancer Hospital of Chinese Medical University, Liaoning Provincial Cancer Institute and Hospital, Shenyang, China; ^3^ Department of Colorectal Surgery, Liaoning Cancer Hospital & Institute, Cancer Hospital of China Medical University, Shenyang, Liaoning, China; ^4^ Department of Medical Image of Liaoning Province, Liaoning Cancer Hospital & Institute, Cancer Hospital of China Medical University, Shenyang, Liaoning, China; ^5^ Department of Radiology, Key Laboratory of Diagnostic Imaging and Interventional Radiology of Liaoning Province, The First Affiliated Hospital of China Medical University, Shenyang, Liaoning, China; ^6^ Key Laboratory of Diagnostic Imaging and Interventional Radiology of Liaoning Province, Department of Radiology, The First Affiliated Hospital of China Medical University, Shenyang, China

**Keywords:** oxygen metabolism, prognosis model, transcriptional regulation, colorectal cancer, hub gene

## Abstract

**Backgroud:**

Oxygen metabolism is an important factor affecting the development of tumors, but its roles and clinical value in Colorectal cancer are not clear. We developed an oxygen metabolism (OM) based prognostic risk model for colorectal cancer and explored the role of OM genes in cancer.

**Methods:**

Gene expression and clinical data obtained from The Cancer Genome Atlas, Clinical Proteomic Tumor Analysis Consortium databases were consider as discovery and validation cohort, respectively. The prognostic model based on differently expressed OM genes between tumor and GTEx normal colorectal tissues were constructed in discovery cohort and validated in validation cohort. The Cox proportional hazards analysis was used to test clinical independent. Upstream and downstream regulatory relationships and interaction molecules are used to clarify the roles of prognostic OM genes in colorectal cancer.

**Results:**

A total of 72 common differently expressed OM genes were detected in the discovery and validation set. A five-OM gene prognostic model including *LRT2*, *ATP6V0E2*, *ODC1*, *SEL1L3* and *VDR* was established and validated. Risk score determined by the model was an independent prognostic according to routine clinical factors. Besides, the role of prognostic OM genes involves transcriptional regulation of MYC and STAT3, and downstream cell stress and inflammatory response pathways.

**Conclusions:**

We developed a five-OM gene prognostic model and study the unique roles of oxygen metabolism in of colorectal cancer

## 1 Introduction

Colorectal cancer has always been the third largest malignant tumor in the world (1). Although the treatment level has been significantly improved in recent years, the morbidity rate remains high and the 5-year survival rate is still low due to the complex and elusive mechanism of cancer formation and development (2, 3). Therefore, finding suitable prognostic markers and new therapeutic targets is still an urgent problem to be solved in the treatment of colorectal cancer

Oxygen (O_2_) is an important catalyst for mitochondria to produce ATP and other intracellular reactions. Hypoxia can induce adaptive responses at multiple cell and body levels to enable individuals to maintain normal metabolism and life activities in a hypoxic environment (4). When in hypoxia environment, cancer cells utilize O_2_-sensing pathways like HIF transcriptional regulators, mTOR and mitochondrial ROS regulation, to overcome oxygen/nutrient deprived microenvironment stresses (5, 6). HIF stabilization and activation are highly responsive to hypoxia and redox stresses, as well as genetic alterations in oncogene or tumor suppressor signaling pathways to support tumor cell survival, growth, and proliferation (5, 7). Some regulators of HIF activity like ROS and cellular ascorbate levels are associated with weaker invasive ability in colorectal cancers (8). A common feature of tumor cells is that even under the normal oxygen condition, increased rates of glycolysis (the “Warburg effect”) which is the critical step for the biosynthesis of ATP and other compounds essential for cell growth and division (6). Additionally, hypoxia has been shown to be associated with therapeutic resistance, including radiation therapy and cytotoxic drugs (9, 10). As an attractive therapeutic target in cancer (11), drugs target on HIFs often lack of specificity on inhibiting subunit (12). Thus, finding credible molecular markers and drug targets related to oxygen metabolism is still challenging.

In recent years, cancer omics research reveals several molecular markers for prognosis monitoring and target therapy of colorectal cancer (13, 14). However, as far as we know, molecular markers related to oxygen metabolism have not been studied in colorectal cancer. A previous study established an eleven gene diagnostic model, and this metadata gene signature had been developed to have an excellent ability to predict diagnosis of TCGA colon cancer patients (15). Another study (16) found that a signature based on 15 metabolites generated from energy supply, macromolecules and oxidative stress has great prognosis potential for colon cancer. The genes significantly correlated to the level of oxygen stress are GPX1, GSTP1, GSR, GSS, GGCT, ANPEP, CAT and ERCC2. Among them, the genes related to oxygen metabolism, such as GPX1, have been included in our gene set. Different from us, the author focused on metabolites, and did not study whether the expression of these genes in tumors was different from normal tissues (16). These studies suggest us that the oxygen metabolism is very likely to have high prognostic and therapeutic value in the colon cancer, but it has not been studied in colorectal cancer so far. So, our research focused on genes related to oxygen metabolism. We found more than 3000 genes related to oxygen metabolism (not just the oxygen metabolism pathway, see methods). Then we determined that the signature of five oxygen metabolism genes, such as VDR, has the highest prognostic potential through DEG analysis, modeling and evaluation of prognosis performance. In order to study the possible mechanism of these genes in colon cancer, we further conducted a detailed functional analysis of each of them to improve the reliability and reference of our research.

In this report, we investigated the expression profile of oxygen metabolism genes, developed and validated a reliable prognostic model of colorectal cancer using differentially expressed oxygen metabolism (OM, **Table 1**) genes. In addition, we set up a protein regulatory network of prognostic genes and explored its potential role in tumorigenesis. This study comprehensively uncovered the prognostic and therapeutic value of oxygen metabolism genes in colorectal cancer patients.

**Table 1:** List of abbreviation used in this paper.

Abbreviation	Definition
OM	oxygen metabolism
TCGA	The Cancer Genome Atlas
GTEx	Genotype-Tissue Expression
DEG	Differential expressed oxygen metabolic gene
MSigDB	Molecular SignaturesDatabase
GSEA	gene set enrichment analysis
OS	overall survival
LASSO	Least absolute shrinkage and selection operator
RS	Risk score
ROC	Receiver operating characteristic curve
AUC	area under the ROC
PPI	protein-protein interaction
TF	transcription factors
K-M	Kaplan-Meier
KEGG	Kyoto Encyclopedia of Genes and Genomes
GO	Gene Ontology
MCODE	Molecular Complex Detection

## 2 Materials and methods

### 2.1 Sample collection

The gene expression data and clinical information of Colorectal cancer patients (n=288) downloaded from TCGA database (https://portal.gdc.cancer.gov/) were used as discovery cohort. The CPTAC-2 prospective data set including gene expression and clinical data of Colorectal cancer patients (n=110) obtained from cBioPortal (https://www.cbioportal.org) were used as validation cohort. Gene expression of Colorectal tissues obtained from GTEx database (https://gtexportal.org/home/)was used as normal control in the downstream analysis (n=253).

### 2.2 Identification of differentially expressed metabolic genes

We first obtained pathways and biological processes from Molecular Signatures Database (MSigDB) C2 curated gene sets on gene set enrichment analysis (GSEA) website. Then, a total of 3524 genes in these gene sets that associated with oxygen metabolism were identified as oxygen metabolism related genes in our study. Differential expressed oxygen metabolic genes (DEG) between tumor and normal samples were analyzed in discovery and validation cohort using ‘limma’ R package, respectively. Genes with FDR< 0.05 and |log_2_(FoldChange)| > 1 were extracted as differentially expressed genes. The “Pheatmap” and “ggplots” package was used to plot heatmaps and volcano maps for DEGs. Venn plots of up- and down-regulated DEGs between discovery and validation cohort were achieved using a Venn online tool (https://bioinformatics.psb.ugent.be/webtools/Venn/)

### 2.3 Construction of the prognostic model

DEGs significantly associated with overall survival (OS) in the entire discovery cohort were identified using univariate Cox proportional hazards regression analyses. A P-value ≤.05 was considered statistically significant. Then, we performed the least absolute shrinkage and selection operator (LASSO) penalty Cox regression analysis to eliminate genes that might overfit the model (Combined-24). Finally, we calculated risk score (RS) for each patient by a linear combination of Cox coefficient and expression of optimal prognostic DEGs identified by multivariate Cox analysis. The risk score calculation formula was as following:


$$\text{RS}=\overset{N}{\underset{1}{\sum }}\left(Ei\times Ci\right)$$

*Ei* and *Ci* represented *i*th gene expression and corresponding coefficient value. N is the number of optimal prognostic DEGs. Patients with RS values greater than the median were defined as high-risk groups, otherwise as low-risk groups. Kaplan-Meier analysis was conducted using the “survival” and “survminer” R package. Receiver operating characteristic curve (ROC) and the “area under the ROC” (AUC) analysis were used to evaluate the performance of the prognostic model.

### 2.4 Validation of the prognostic model

We used validation cohort (CPTAC) to verify the prognostic risk model. RS of each patient in validation cohort was calculated using formula mentioned above based prognostic DEGs and coefficient identified in discovery cohort. Survival and ROC analysis were used to validate the performance of prognostic risk model.

### 2.5 Independent prognostic value of prognostic model

To assess the independent prognostic value of oxygen metabolic gene-based risk models in colorectal cancer, we performed both univariate and multivariate analyses of prognostic factors using Cox proportional hazards regression. Age, gender, pathological stage and TNM stage were treated as covariates. Factors with p value< 0.05 in both univariate and multivariate Cox analysis were defined as independent prognostic indicators.

### 2.6 Protein-protein interaction network based on prognostic genes

We constructed the PPI network of prognostic oxygen-metabolic genes using the PathwayCommon (https://www.pathwaycommons.org/) PPI database. Analysis of functional interactions between proteins was performed in order to elucidate the potential roles of prognostic genes in colorectal cancer tumorigenic process. The PPI networks were visualized using the Cytoscape software.

### 2.7 Hub prognostic genes and their upstream transcription factors

The hub genes were identified using DMNC, MNC, Degree, EPC, BottleNeck, EcCentricity, Closeness, Radiality, Betweenness, Stress and ClusteringCoefficient algrithms with Cytoscape’s plug-in cytoHubba in the PPI network. Then, we obtained all possible transcription factors (TFs) of hub gene from ChiIP seq experimental data of human samples in the ENCODE project, and identified upstream TFs that play a role in colon cancer by calculating the expression correlation between these TFs and hub genes in our tumor samples. The correlationship of gene expression between hub genes and their TFs was conducted using the spearman method. The most relevant TF-hub gene relationship was shown by scatter plots.

## 3 Results

### 3.1 Identification of differentially expressed and survival-related OM genes

**Figure 1:**
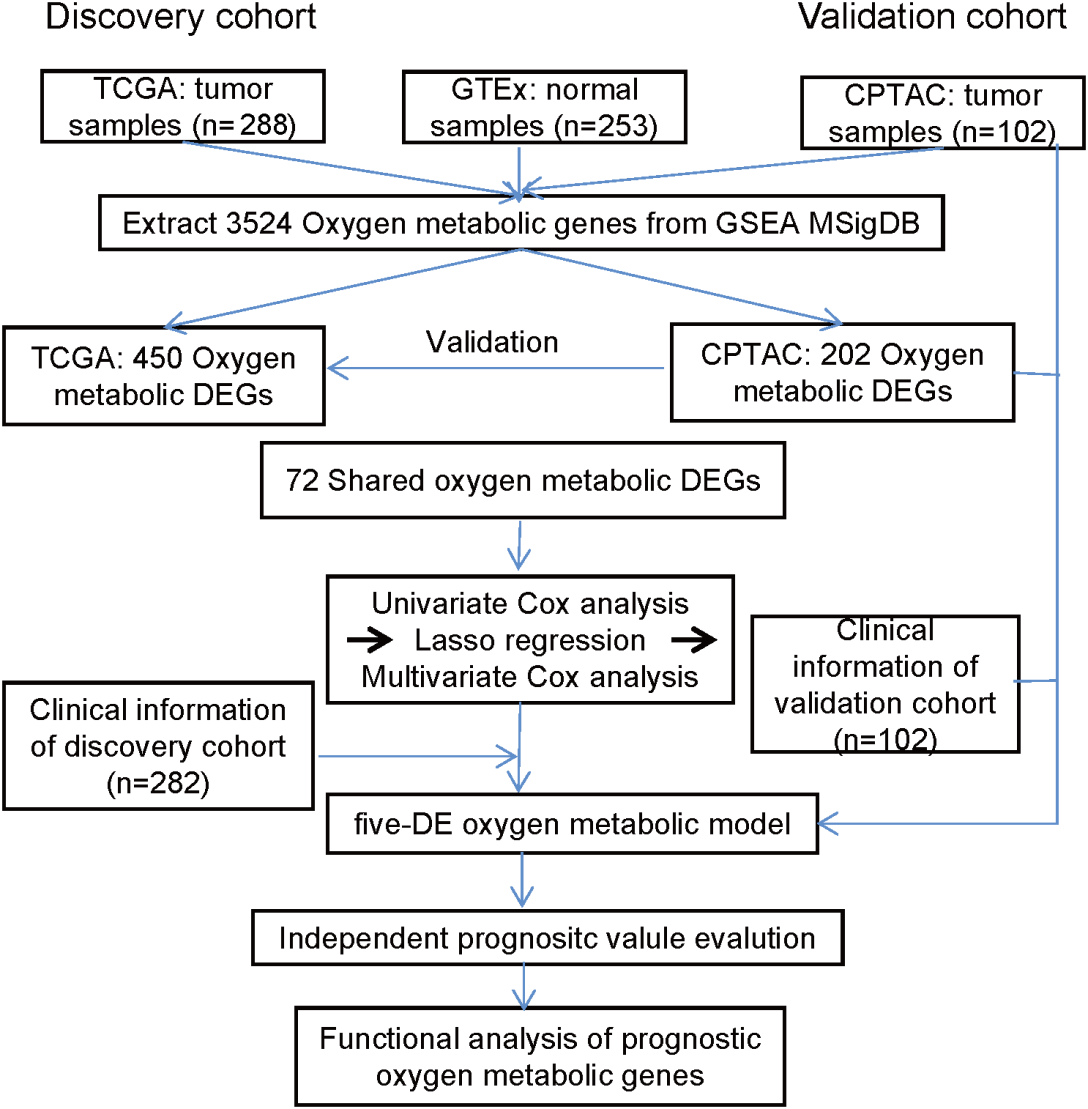
The workflow of this study. The prognostic model based on oxygen metabolism genes was established and validated in two independent CRC cohorts, and the roles of prognostic genes in CRC was further analyzed.

**Figure 2:**
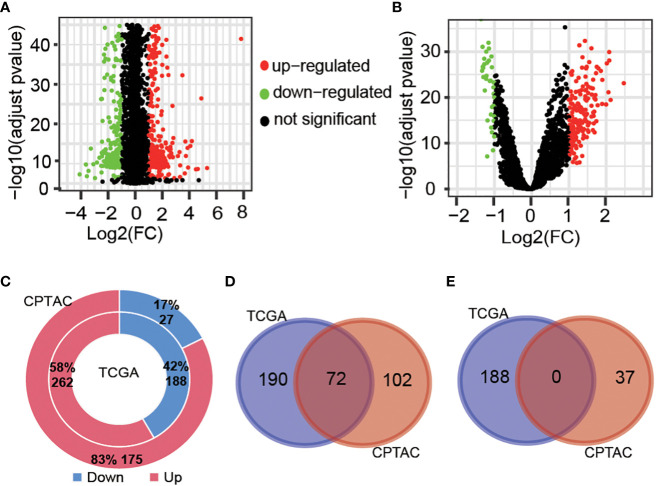
Different expressed gene analysis between tumor and normal samples. Volcanic map showing the difference of gene levels between tumor and normal samples in the TCGA cohort **(A)** and CPTAC cohort **(B)**. Genes with |Log2(FC)| >1 and adjust pvalue<0.01 were defined as different expressed genes. **(C)**, Proportion and number of significantly up-regulated and down-regulated genes obtained from two cohort samples. Venn diagrams of up-regulated genes **(D)** and down-regulated genes **(E)** in TCGA and CPTAC samples.

The workflow of this study is shown in **Figure 1**. Tumor samples (n=288) obtained from the TCGA database were regarded as the discovery cohort, while tumor samples (n=102) obtained from the CPTAC project were regarded as validation cohort. We compared expression levels of 3524 oxygen metabolic genes between tumor and normal samples in discovery set and validation set, respectively. The distributions of all genes including identified DEGs according to the two dimensions of -log_10_(FDR) and log_2_(FoldChange) were displayed by volcano maps (**Figures 2A, B**). It was found that there were 262 up-regulated and 188 down-regulated genes in the discovery set, 175 up-regulated and 27 down-regulated genes in the validation set (**Figure 2C**). To obtained the more reliable prognostic gene signature, we established the prognostic model with 72 DEGs up-regulated in both the discovery set and the validation set (**Figures 1**, **2D**). We didn't obtaied reliable down-regulated DEGs which were identified in the discovery and validation cohorts **Figure 2E**)

Univariate Cox regression analysis revealed that 95 OM DEGs were significantly (*P*<.05) associated with OS in the discovery cohort. Among them, 73 DEGs were associated with good OS, while 22 DEGs were associated with bad OS.

### 3.2 Construction of a five-OM gene prognostic model

Based on the discovery cohort, we obtained eight candidate prognostic OM genes using Lasso Cox regression analysis. Then, we acquired five optimal genes, including *FLRT2* (Fibronectin Leucine Rich Transmembrane Protein 2), *ATP6V0E2* (ATPase H+ Transporting V0 Subunit E2), *ODC1* (Ornithine Decarboxylase 1), *SEL1L3* (SEL1L Family Member 3) and *VDR* (Vitamin D Receptor). Four of these genes were high hazard genes, and one gene (SEL1L3) was low hazard gene, and all these genes were up-regulated DE genes (**Table 2**). The risk score of each tumor sample was calculated as follows: risk score = (1.0232× *FLRT2*_exp_) + (1.0046 ×  *ATP6V0E2*_exp_) + (0.9806 ×  *SEL1L3*_exp_) + (1.0015 × *ODC1*_exp_) +  (1.0493 × *VDR*_exp_).

**Table 2:** Five prognostic oxygen-metabolic genes.

Genes	HR	CI(95%) Lower	CI(95%) Upper	pvalue
FLRT2	1.0232	1.0008	1.0461	0.0424
ATP6V0E2	1.0046	1.0015	1.0076	0.0037
ODC1	1.0015	1.0004	1.0026	0.0059
SEL1L3	0.9806	0.9690	0.9923	0.0012
VDR	1.0493	1.0262	1.0731	0.0000

**Figure 3:**
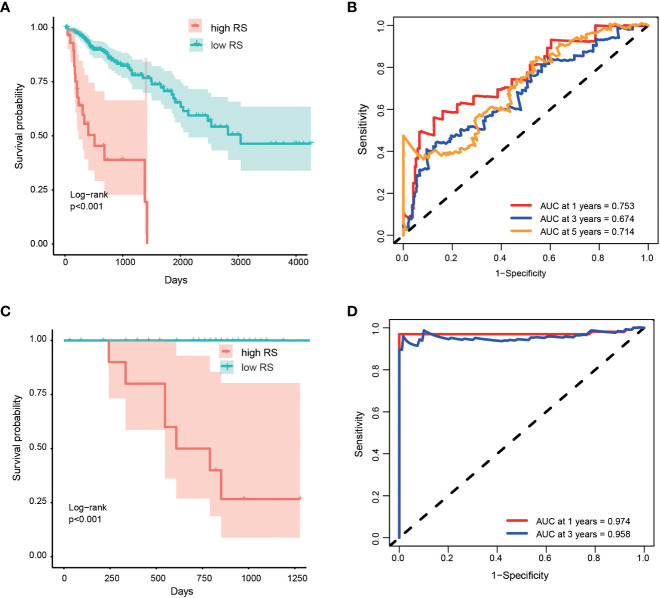
Performance of prognostic risk model in discovery and validation cohort. Kaplan-Meier curves shown the overall survive among patients classified into high- and low- RS groups in discovery **(A)** and validation **(C)** cohorts. The difference of survival time between the two groups was tested by log rank method. ROC curves and their AUC value shown the performance of the prognostic risk model in predicting the one, three and five years survive time in discovery **(B)** and validation **(D)** cohort.

Based on the optimized risk score threshold, all colorectal cancer patients of discovery cohort were divided into a high-risk group (n = 30) and a low-risk group (n = 252). The K-M survival analysis shown those OS times of high-risk patients were significantly longer than that of low-risk patients (p< 0.001) (**Figure 3A**). The median survival time of patients in the high-risk group was shorter than 5 years, while that of patients in the low-risk group was longer than 10 years. From the perspective of survival rate, the 1-year, 3-year and 5-year survival rates of the high-risk group were only 72%, 63% and 35% respectively, while the corresponding survival rates of the low-risk group reached 91%, 85% and 71% respectively. In addition, the AUC values of the five-OM gene prediction model were 0.753, 0.674, and 0.714 when predicting one -, three -, and five-year OS, respectively (**Figure 3B**). To find out whether all 5 prognosis OM genes are associated with advanced stages and therefore are associated with worse prognosis, we analyzed the OS time of patients with high- and low-RS from tumor stage I, II, III and IV. Results indicated that the OS time of patients with high RS was significantly shorter than that of patients with low RS in stage II and IV, which proved the prognostic effectiveness of our 5-OM gene signature in these two stages. But in stage I and III, there was no significant difference in the OS time of patients with high- and low-RS, suggesting the prognostic limitations of the model in these two stages (**Figure S1**).

### 3.3 Validation of the prognostic model

We validated the performance of the model using the validation cohort. Patients in validation cohort were divided into high- and low- risk groups based on RS threshold determined in discovery cohort. Results indicated that 12 patients and 90 patients were categorized as high- and low- risk groups, respectively. K-M survival curves were significant different between the two risk groups (p < 0.001) (**Figure 3C**) and the AUC values at 1- and 3-year were 0.974 and 0.958 in the validation cohort, respectively (**Figure 3D**). At the same time, the RSs of patients in the high-risk group were higher than those in the low-risk group, which proved that the model had a good performance in the prognosis evaluation and monitoring of colorectal cancer.

### 3.4 Independent prognostic ability of prognostic model

To assess whether the RS determined by five oxygen-metabolic prognostic model is an independent prognostic indicator for patients, we carried out a univariate Cox analysis to assess the impact of risk scores and clinicopathological parameters on prognosis, such as age, gender, histological type, longest dimension, pathological stage and so on. We found that the longest dimension, lymphatic invasion, pathological stage and risk score were associated with poor outcomes of prognosis in patients (**Table 3**). Therefore, these characteristics were included in a multivariate Cox regression analysis, which indicated that age, pathological stage and the risk score estimated based the prognostic model was an independent prognostic factor for colorectal cancer (**Table 3**). This result indicates that there is significant potential for these oxygen-metabolic genes to predict the prognosis outcome of patients with the colorectal cancer.

**Table 3:** Cox regression analyses of RS and clinicopathological parameters related to prognosis in CRC patients.

Variables	OS			
	Univariate analysis		Multivariate analysis	
	HR (95% CI)	*P*	HR (95% CI)	*P*
Gender(male/female)	1.160 (0.495-2.717)	.733		
Age (>70/≤70)	1.013 (0.990-1.038)	.072	2.205 (1.200-4.052)	.011
Longest_dimension (>1 cm/≤ 1 cm)	2.275 (1.296-3.994)	.004	1.835 (1.295-3.650)	.067
Histological type Adenocarcinoma Mucinous	1.323 (0.526-3.328)	.552		
Lymphatic_invasion (Yes/No)	1.000 (1.081-3.281)	.025	1.984 (1.043-3.601)	.051
pathologic_stage (I/II/III/IV)	1.516 (1.156-1.987)	.003	1.962 (1.196-3.684)	.031
number_of_lymphnodes	1.017 (0.913-1.038)	.242		
postoperative_rx_tx	1.032 (0.826-1.328)	.552		
Risk score (high/low)	2.497 (1.380-4.516)	.002	1.896 (1.004-3.481)	.021

### 3.5 Transcriptional regulation of prognostic oxygen-metabolic genes

**Figure 4:**
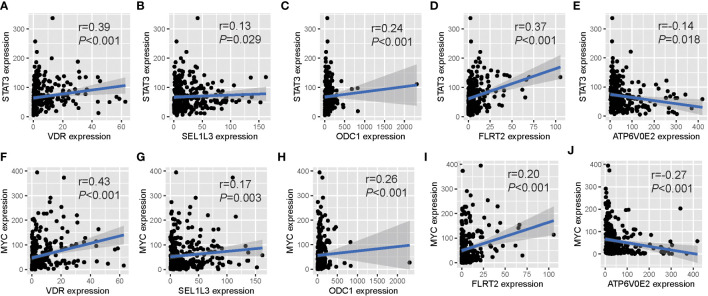
Expression relationships between prognostic OM genes and upstream TFs. Significant correlations of gene expression between five prognostic OM genes and their common upstream TFs: STAT3 **(A-E)** and MYC **(F-J)**.

We investigated the regulatory relationships between TFs and prognostic genes. Firstly, we obtained upstream TFs of each gene from the ChIP-Seq experiment in the ENCODE project (https://www.encodeproject.org). Then, we analyzed expression correlation of between TFs and genes in TCGA tumor samples to validate the TF regulation *in vivo*. We found that 11 cancer-related TFs including CTBP2, E2F1, EP300, ETS1, FOS, JUN, MYC, RELA, STAT1, STAT3 and TCF7L2 were significantly correlated with our prognostic genes. Among them,*STAT3* and *MYC* were significantly correlated with all prognostic genes, in which positively correlated with *FLRT2*, *ODC1*, *SEL1L3* and *VDR*, and negatively correlated with *ATP6V0E2* (**Figure 4**).These results indicates that the prognostic genes we screened are important downstream molecules of classic cancer driver genes like STAT3 and MYC.

### 3.6 Functional analysis of prognostic oxygen-metabolic genes

**Figure 5:**
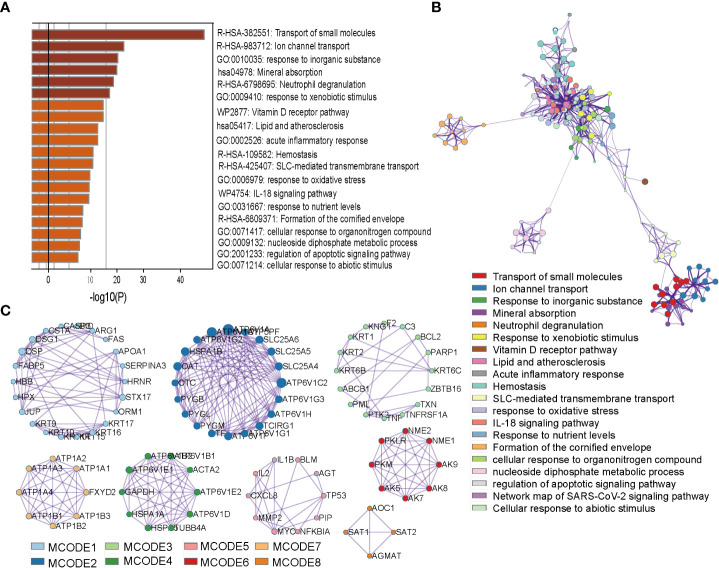
Enrichment and interact network of prognostic OM genes participating. **(A)** Bar graph of terms enriched across prognostic OM genes and their interacting genes. **(B)** Network of enriched terms colored by cluster ID, where nodes that share the same cluster ID are typically close to each other. **(C)**, Densely connected network components identified by the Molecular Complex Detection (MCODE) algorithm.

In order to study the possible function and mechanism of prognostic oxygen metabolic genes in colorectal cancer, we screened KEGG cancer pathway genes that can interact with prognostic genes, and then used the metascape to analyze the gene ontology (GO) and pathway enrichment of interacting genes It was found that the interaction genes were significantly enriched in GO terms including “response to inorganic substance”, “response to xenobiotic stimulus”, “response to oxidative stress”, and the enriched pathways were “transport of small molecules”, “ion channel transport”, “mineral absorption” and so on (**Figure 5A**). To further capture the relationships between these enriched terms, we constructed a network diagram using Metascape analysis. Spots represented GO terms or pathways. Larger and connected points represented the presence of more similar genes between the terms or pathways. The “Transport of small molecules” pathway contained many genes participating in Ion channel transport, while the “response to oxidative stress” gene set contained many genes participating in cell stress and inflammatory response terms or pathways (**Figure 5B**). In addition, the PPI network showed a relationship between different genes and proteins in two sub-modules (**Figure 5B**). The “Fluid shear stress and atherosclerosis” sub-module seeded by the MYC included IL1B, TP53, PIP, MYC, IL2, NFKBIA. AGT, CXCL8, BLM and MMP2, which can identify the structural components of the extracellular matrix to provide tensile strength; the “extracellular matrix organization” sub-module included SPP1, IGFBP4, GAS6, MXRA8, and SPARCL1, which play a central role in vascular biology; the “Signaling by Interleukins” sub-module seeded by the F2 included KNG1, KRT6B, PARP1, TNFRSF1A. KRT2, ZBTB16. ABCB1, BCL2, KRT1. PML, C3, TXN, KRT6C, F2, PTK2, TNF, which could enable HIF-mediated inflammatory response during cancer development (**Figure 5C**).

## 4 Discussion

The colorectal cancer is the malignant tumor with the third highest incidence rate and the second highest mortality rate in the world (17). In 2020 alone, 1.9 million people were diagnosed, of which 0.9 million died (1). Several prognostic models have been established in colorectal cancer (18–20). However, there are some drawbacks in currently existing prognostic risk models of colorectal cancer. Firstly, the sample sizes are insufficient to represent the whole disease population, which makes the risk score summarized from the level of gene expression uncertain for clinical personalized prognosis. Because patients’ risk scores often depend on other samples used for normalization data (21). Secondly, most of the existing prognostic models are based on the expression of all genes. Because there is no focus on a certain biological process, it is difficult to study the relationship between prognostic genes and explain the biological mechanism behind the prognostic model. Thirdly, those prognostic models based on non-coding genes or omic modification features have the problem of high detection cost and easy to produce bias during applications (18, 19). Several studies have reported the roles of the oxygen metabolism in tumorigenesis and development of cancer (6, 7, 22). In this study, we built and validated a prognostic model using five oxygen metabolic genes higher expressed in tumor samples. The survival time of high-risk patients predicted by the model is significantly shorter than that of low-risk patients. At the same time, the model is good in predicting the survival time of patients stratified by survival time. Additionally, Multivariate cox analysis indicates that the model can predict overcome of CRC patients independently when mixed with age, stage, pathological grade and other factors. So, we demonstrate the important but long neglected clinical prognostic value of OM genes in CRC.

Five oxygen metabolic genes named *FLRT2*, *ATP6V0E2*, *ODC1*, *SEL1L3* and *VDR* were prognostic genes determined by the prognostic model. The expression of these genes was all higher in tumor than in normal tissues, which might play important roles in CRC progression and contribute to the early diagnosis. The *FLRT2* is highly expressed in tumor neovascularization and forms abnormal endothelial adhesion to prevent oxidative stress of cells. Its expression level is positively correlated with the short-term survival in the advanced colorectal cancer (23). The expression of FLRT2 is dependent on oxidative stress but not on VEGF (24), indicates that FLRT2 may play an important role in oxygen metabolism. The *ATP6V0E2* might promote cancer cell death and tumor suppression with high levels of ROS (reactive oxygen species) through inhibition of lysosomal function (25). *ODC1* activity is frequently elevated in cancer through deregulation of MYC, resulting in higher polyamine content to support rapid tumor cell proliferation (26). A study has shown that the expression of *SEL1L3* is elevated in endometrial cancer. In white patients with low mutation load, the expression level of this gene is related to the patient’s recurrence free productivity and is considered as a potential driver and tumor marker of endometrial cancer (27).. SEL1L3 was also positively correlated with reactive oxygen species such as hydrogen peroxide (28). An elegant series of studies found that the VDR signaling affect tumor development by the delicate interplay with E-cadherin and the Wnt signaling pathway (29–32). All five identified prognostic genes are proved to play certain roles in tumors, which prove the reliability of our prognosis model in biological sense.

Transcriptional regulation and functional analysis gives us an in-depth understanding of the possible molecular mechanisms behind the prognostic model. An upstream regulatory factor MYC and STAT3 are constitutively activated in many cancers and plays a pivotal role in tumor growth and metastasis by regulating cell proliferation, invasion, migration, and angiogenesis (33–36). Myc promotes the transcription of STAT3 (37), then hypoxic stress markedly increased phosphorylated STAT3 level in a time-dependent fashion, and activated STAT3 was translocated into the nucleus (38). After that, the lysosomal activation was blocked by down-regulating ATP6V0E2 through the JAK2-STAT3-VEGFA singling pathway, to inhibit cell apoptosis in human colon cancer (25). SEL1L3 which is a target of transcript factor STAT3 and MYC plays important roles in oxygen metabolism related pathway “ SUNG_METASTASIS_ STROMA_UP”. Downstream interaction genes are mainly enriched in angiogenesis and inflammatory response in tumors. Angiogenesis is a critical step in cancer progression and is considered one of the hallmarks of cancer, and validated as an independent prognostic factor and the culprit of drug resistance in a variety of solid malignancies including colorectal cancer (39–42).

This study has several advantages. Firstly, we constructed a prognostic model based on DE OM genes in colorectal cancer for the first time. Secondly, the prognostic model was proved to be accurate and reliable using an independent cohort. Thirdly, the risk score determined by the model could be used as an independent prognostic index in predicting OS. Finally, we found that five prognostic OM genes regulate angiogenesis and inflammatory response in colorectal cancer. However, in our study, the RNA-seq data was used to obtain the gene expression levels in tumor and normal tissues, and determined the risk thresholds of patients with a prognosis model based on expression levels of the gene signature. Studies (21, 43, 44) have shown that RNA-seq data set-generated risk thresholds cannot be directly applied to independent microarray data sets because the gene expression levels are sensitive to systematic biases of microarray measurements owing to batch effects and platform differences. We also did not verify our prognostic risk model at the protein level in an independent cohort. So, we have started to collect patients and CRC samples so that we can obtain the protein levels by IHC and verify the risk model in an independent cohort in the future.

## Data availability statement

The datasets presented in this study can be found in online repositories. The names of the repository/repositories and accession number(s) can be found in the article/**Supplementary Material**.

## Author contributions

YY conceptualized the study and designed the research. YY, XQ and ZZ organized all the studies. YY, ZZ and BM completed data analysis and interpretation. YY, ZZ, XQ, PJ and SM wrote and revised the article. All authors contributed to the article and approved the submitted version.

## References

[1] Xi Y Xu P. Global colorectal cancer burden in 2020 and projections to 2040. Trans Oncol (2021) 14:101174. doi: 10.1016/j.tranon.2021.101174 PMC8273208 34243011

[2] Center MM Jemal A Smith RA Ward E. Worldwide variations in colorectal cancer. CA Cancer J Clin (2009) 59:366–78. doi: 10.3322/caac.20038 19897840

[3] Edwards BK Ward E Kohler BA Eheman C Zauber AG Anderson RN. Annual report to the nation on the status of cancer, 1975-2006, featuring colorectal cancer trends and impact of interventions (risk factors, screening, and treatment) to reduce future rates. Cancer (2010) 116:544–73. doi: 10.1002/cncr.24760 PMC3619726 19998273

[4] Lee KE Simon MC. SnapShot: Hypoxia-inducible factors. Cell (2015) 163:1288–1288.e1. doi: 10.1016/j.cell.2015.11.011 26590427

[5] Qiu B Simon MC. Oncogenes strike a balance between cellular growth and homeostasis. Semin Cell Dev Biol (2015) 43:3–10. doi: 10.1016/j.semcdb.2015.08.005 26277544 PMC4662909

[6] Pavlova NN Thompson CB. The emerging hallmarks of cancer metabolism. Cell Metab (2016) 23:27–47. doi: 10.1016/j.cmet.2015.12.006 26771115 PMC4715268

[7] Ratcliffe PJ. Oxygen sensing and hypoxia signalling pathways in animals: the implications of physiology for cancer. J Physiol (2013) 591:2027–42. doi: 10.1113/jphysiol.2013.251470 PMC3634517 23401619

[8] Kuiper C Dachs GU Munn D Currie MJ Robinson BA Pearson JF. Increased tumor ascorbate is associated with extended disease-free survival and decreased hypoxia-inducible factor-1 activation in human colorectal cancer. Front Oncol (2014) 4:10. doi: 10.3389/fonc.2014.00010 24551593 PMC3912592

[9] Brizel DM Scully SP Harrelson JM Layfield LJ Bean JM Prosnitz LR. Tumor oxygenation predicts for the likelihood of distant metastases in human soft tissue sarcoma. Cancer Res (1996) 56:941–3. 8640781

[10] Hockel M Knoop C Schlenger K Vorndran B Baussmann E Mitze M. Intratumoral pO2 predicts survival in advanced cancer of the uterine cervix. Radiother Oncol J Eur Soc Ther Radiol Oncol (1993) 26:45–50. doi: 10.1016/0167-8140(93)90025-4 8438086

[11] Bertout JA Patel SA Simon MC. The impact of O2 availability on human cancer. Nat Rev Cancer (2008) 8:967–75. doi: 10.1038/nrc2540 PMC3140692 18987634

[12] Semenza GL. HIF-1 inhibitors for cancer therapy: from gene expression to drug discovery. Curr Pharm Des (2009) 15:3839–43. doi: 10.2174/138161209789649402 19671047

[13] Kohne CH. Successes and limitations of targeted cancer therapy in colon cancer. Prog Tumor Res (2014) 41:36–50. doi: 10.1159/000356436 24727985

[14] Yang C Zhang Y Xu X Li W. Molecular subtypes based on DNA methylation predict prognosis in colon adenocarcinoma patients. Aging (Albany NY) (2019) 11:11880–92. doi: 10.18632/aging.102492 PMC6949097 31852837

[15] Zuo D Li C Liu T Yue M Zhang J Ning G. Construction and validation of a metabolic risk model predicting prognosis of colon cancer. Sci Rep (2021) 11:6837. doi: 10.1038/s41598-021-86286-z 33767290 PMC7994414

[16] Qiu Y Cai G Zhou B Li D Zhao A Xie G. A distinct metabolic signature of human colorectal cancer with prognostic potential. Clin Cancer Res an Off J Am Assoc Cancer Res (2014) 20:2136–46. doi: 10.1158/1078-0432.CCR-13-1939 PMC5902798 24526730

[17] Keum N Giovannucci E. Global burden of colorectal cancer: emerging trends, risk factors and prevention strategies. Nat Rev Gastroenterol Hepatol (2019) 16:713–32. doi: 10.1038/s41575-019-0189-8 31455888

[18] Luo H Zhao Q Wei W Zheng L Yi S Li G. Circulating tumor DNA methylation profiles enable early diagnosis, prognosis prediction, and screening for colorectal cancer. Sci Transl Med (2020) 12. doi: 10.1126/scitranslmed.aax7533 31894106

[19] Zeng H Xu Y Xu S Jin L Shen Y Rajan KC. Construction and analysis of a colorectal cancer prognostic model based on N6-Methyladenosine-Related lncRNAs. Front Cell Dev Biol (2021) 9:698388. doi: 10.3389/fcell.2021.698388 34490250 PMC8417314

[20] Liu C Wang T Yang J Zhang J Wei S Guo Y. Distant metastasis pattern and prognostic prediction model of colorectal cancer patients based on big data mining. Front Oncol (2022) 12:878805. doi: 10.3389/fonc.2022.878805 35530362 PMC9074728

[21] Qi L Chen L Li Y Qin Y Pan R Zhao W. Critical limitations of prognostic signatures based on risk scores summarized from gene expression levels: a case study for resected stage I non-small-cell lung cancer. Brief Bioinform (2016) 17:233–42. doi: 10.1093/bib/bbv064 26254430

[22] Semenza GL. HIF-1 mediates metabolic responses to intratumoral hypoxia and oncogenic mutations. J Clin Invest (2013) 123:3664–71. doi: 10.1172/JCI67230 PMC3754249 23999440

[23] Ando T Tai-Nagara I Sugiura Y Kusumoto D Okabayashi K Kido Y. Tumor-specific interendothelial adhesion mediated by FLRT2 facilitates cancer aggressiveness. J Clin Invest (2022) 132 (6). doi: 10.1172/JCI153626 PMC8920344 35104247

[24] Jauhiainen S Laakkonen JP Ketola K Toivanen PI Nieminen T Ninchoji T. Axon guidance-related factor FLRT3 regulates VEGF-signaling and endothelial cell function. Front Physiol (2019) 10:224. doi: 10.3389/fphys.2019.00224 30930791 PMC6423482

[25] Sun X Shu Y Yan P Huang H Gao R Xu M. Transcriptome profiling analysis reveals that ATP6V0E2 is involved in the lysosomal activation by anlotinib. Cell Death Dis (2020) 11:702. doi: 10.1038/s41419-020-02904-0 32839434 PMC7445181

[26] Hogarty MD Norris MD Davis K Liu X Evageliou NF Hayes CS. ODC1 is a critical determinant of MYCN oncogenesis and a therapeutic target in neuroblastoma. Cancer Res (2008) 68:9735–45. doi: 10.1158/0008-5472.CAN-07-6866 PMC2596661 19047152

[27] Mei Y Chen MM Liang H Ma L. A four-gene signature predicts survival and anti-CTLA4 immunotherapeutic responses based on immune classification of melanoma. Commun Biol (2021) 4:383. doi: 10.1038/s42003-021-01911-x 33753855 PMC7985195

[28] Bekeschus S Liebelt G Menz J Singer D Wende K Schmidt A. Cell cycle-related genes associate with sensitivity to hydrogen peroxide-induced toxicity. Redox Biol (2022) 50:102234. doi: 10.1016/j.redox.2022.102234 35063803 PMC8783094

[29] Ferrer-Mayorga G Gomez-Lopez G Barbachano A Fernandez-Barral A Pena C Pisano DG. Vitamin d receptor expression and associated gene signature in tumour stromal fibroblasts predict clinical outcome in colorectal cancer. Gut (2017) 66:1449–62. doi: 10.1136/gutjnl-2015-310977 PMC5530491 27053631

[30] Alvarez-Diaz S Valle N Garcia JM Pena C Freije JM Quesada V. Cystatin d is a candidate tumor suppressor gene induced by vitamin d in human colon cancer cells. J Clin Invest (2009) 119:2343–58. doi: 10.1172/JCI37205 PMC2719930 19662683

[31] Pendas-Franco N Garcia JM Pena C Valle N Palmer HG Heinaniemi M. DICKKOPF-4 is induced by TCF/beta-catenin and upregulated in human colon cancer, promotes tumour cell invasion and angiogenesis and is repressed by 1alpha,25-dihydroxyvitamin D3. Oncogene (2008) 27:4467–77. doi: 10.1038/onc.2008.88 18408752

[32] Palmer HG Larriba MJ Garcia JM Ordonez-Moran P Pena C Peiro S. The transcription factor SNAIL represses vitamin d receptor expression and responsiveness in human colon cancer. Nat Med (2004) 10:917–9. doi: 10.1038/nm1095 15322538

[33] Dang CV. MYC on the path to cancer. Cell (2012) 149:22–35. doi: 10.1016/j.cell.2012.03.003 22464321 PMC3345192

[34] Wong KE Ngai SC Chan KG Lee LH Goh BH Chuah LH. Curcumin nanoformulations for colorectal cancer: A review. Front Pharmacol (2019) 10:152. doi: 10.3389/fphar.2019.00152 30890933 PMC6412150

[35] Nguyen AV Wu YY Lin EY. STAT3 and sphingosine-1-phosphate in inflammation-associated colorectal cancer. World J Gastroenterol WJG (2014) 20:10279–87. doi: 10.3748/wjg.v20.i30.10279 PMC4130835 25132744

[36] Wang SW Sun YM. The IL-6/JAK/STAT3 pathway: potential therapeutic strategies in treating colorectal cancer (Review). Int J Oncol (2014) 44:1032–40. doi: 10.3892/ijo.2014.2259 24430672

[37] Barre B Vigneron A Coqueret O. The STAT3 transcription factor is a target for the myc and riboblastoma proteins on the Cdc25A promoter. J Biol Chem (2005) 280:15673–81. doi: 10.1074/jbc.M413203200 15677471

[38] Kang SH Yu MO Park KJ Chi SG Park DH Chung YG. Activated STAT3 regulates hypoxia-induced angiogenesis and cell migration in human glioblastoma. Neurosurgery (2010) 67:1386–95. doi: 10.1227/NEU.0b013e3181f1c0cd 20871442

[39] Holash J Maisonpierre PC Compton D Boland P Alexander CR Zagzag D. Vessel cooption, regression, and growth in tumors mediated by angiopoietins and VEGF. Science (1999) 284:1994–8. doi: 10.1126/science.284.5422.1994 10373119

[40] Jacobsen J Rasmuson T Grankvist K Ljungberg B. Vascular endothelial growth factor as prognostic factor in renal cell carcinoma. J Urol (2000) 163:343–7. doi: 10.1016/S0022-5347(05)68049-4 10604387

[41] Maeda K Chung YS Ogawa Y Takatsuka S Kang SM Ogawa M. Prognostic value of vascular endothelial growth factor expression in gastric carcinoma. Cancer (1996) 77:858–63. doi: 10.1002/(SICI)1097-0142(19960301)77:5<858::AID-CNCR8>3.0.CO;2-A 8608475

[42] Carmeliet P Jain RK. Angiogenesis in cancer and other diseases. Nature (2000) 407:249–57. doi: 10.1038/35025220 11001068

[43] Leek JT Scharpf RB Bravo HC Simcha D Langmead B Johnson WE. Tackling the widespread and critical impact of batch effects in high-throughput data. Nat Rev Genet (2010) 11:733–9. doi: 10.1038/nrg2825 PMC3880143 20838408

[44] Geman D d'Avignon C Naiman DQ Winslow RL. Classifying gene expression profiles from pairwise mRNA comparisons. Stat Appl Genet Mol Biol 3 (2004). doi: 10.2202/1544-6115.1071 PMC1989150 16646797

