# Supplementary material for: Effective oxygen metabolism-based prognostic signature for colorectal cancer
Source: Front Oncol. 2023 Feb 9;13:1072941. doi: 10.3389/fonc.2023.1072941 (PMC9947833; doi:10.3389/fonc.2023.1072941)
Supplement: Supplementary file 1 [file Image_1.pdf]

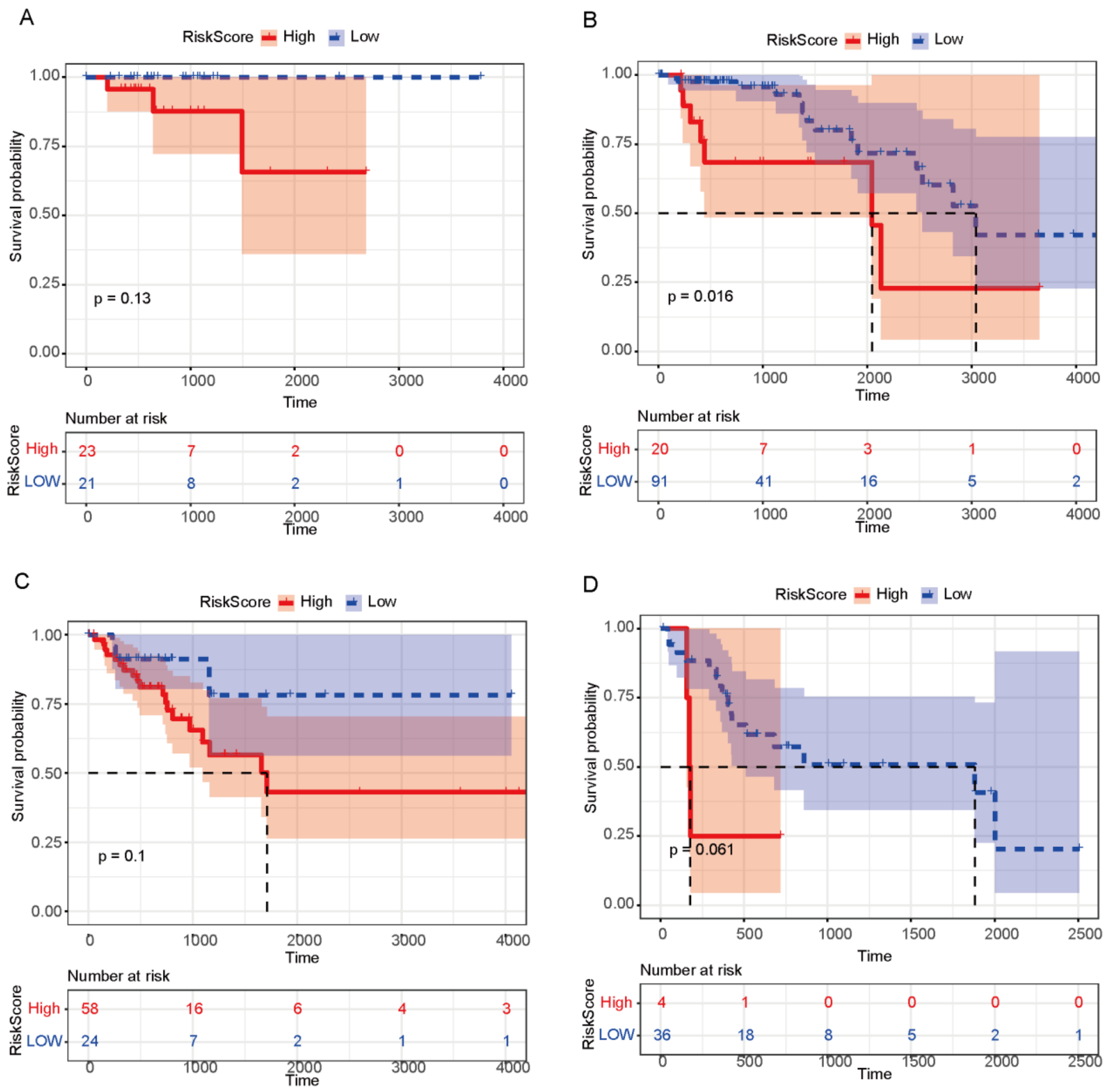

Figure S1. K-M curves show the overall survival among patients classified into high- and low-RS groups in different tumor stages of the TCGA. Tumor stages of patients in (A), (B), (C), and (D) are I, II, III, and IV, respectively. The significance of the difference in survival probability was obtained by log-rank test.
